# CRISPOR: intuitive guide selection for CRISPR/Cas9 genome editing experiments and screens

**DOI:** 10.1093/nar/gky354

**Published:** 2018-05-14

**Authors:** Jean-Paul Concordet, Maximilian Haeussler

**Affiliations:** 1 Muséum national d’Histoire naturelle, INSERM U1154, CNRS UMR 7196, Sorbonne Universités, 43 rue Cuvier, Paris F-75231, France; 2 Genomics Institute, University of California at Santa Cruz, 1156 High Street, MS CBSE, Santa Cruz, CA 95060, USA

## Abstract

CRISPOR.org is a web tool for genome editing experiments with the CRISPR–Cas9 system. It finds guide RNAs in an input sequence and ranks them according to different scores that evaluate potential off-targets in the genome of interest and predict on-target activity. The list of genomes is continuously expanded, with more 150 genomes added in the last two years. CRISPOR tries to provide a comprehensive solution from selection, cloning and expression of guide RNA as well as providing primers needed for testing guide activity and potential off-targets. Recent developments include batch design for genome-wide CRISPR and saturation screens, creating custom oligonucleotides for guide cloning and the design of next generation sequencing primers to test for off-target mutations. CRISPOR is available from http://crispor.org, including the full source code of the website and a stand-alone, command-line version.

## INTRODUCTION

Genome editing with the CRISPR–Cas system is impacting all fields of life sciences. While the technique is comparatively simple, cleavage specificity and efficiency depend primarily on the choice of the CRISPR guide sequence. To help with this step, various web tools find potential guide RNAs in an input sequence and rank them according to off-target and on-target predictions (1–3). We initially developed CRISPOR to help with guide RNA selection in genomes from non-conventional model organisms for which no tools were available yet. While we were developing the website, many design criteria were published, so we compared them, published the comparison (4) and designed CRISPOR such that it recommends scores that are most accurate for a particular assay. Since then, CRISPOR has gained thousands of users and we have added genomes and new features based on their feedback.

Apart from supporting many genomes and predictive scoring models, another distinguishing feature of CRISPOR is that it tries to help with cloning, expressing and validating of guide sequences. Overlapping oligonucleotides, primers for target validation and relevant restriction enzymes are displayed, can be downloaded as tables and in a few cases we link to the respective wet-lab protocols. In the following, we give a quick overview of the software and then describe changes made last year in more detail.

### The typical CRISPOR workflow

**Figure 1.**
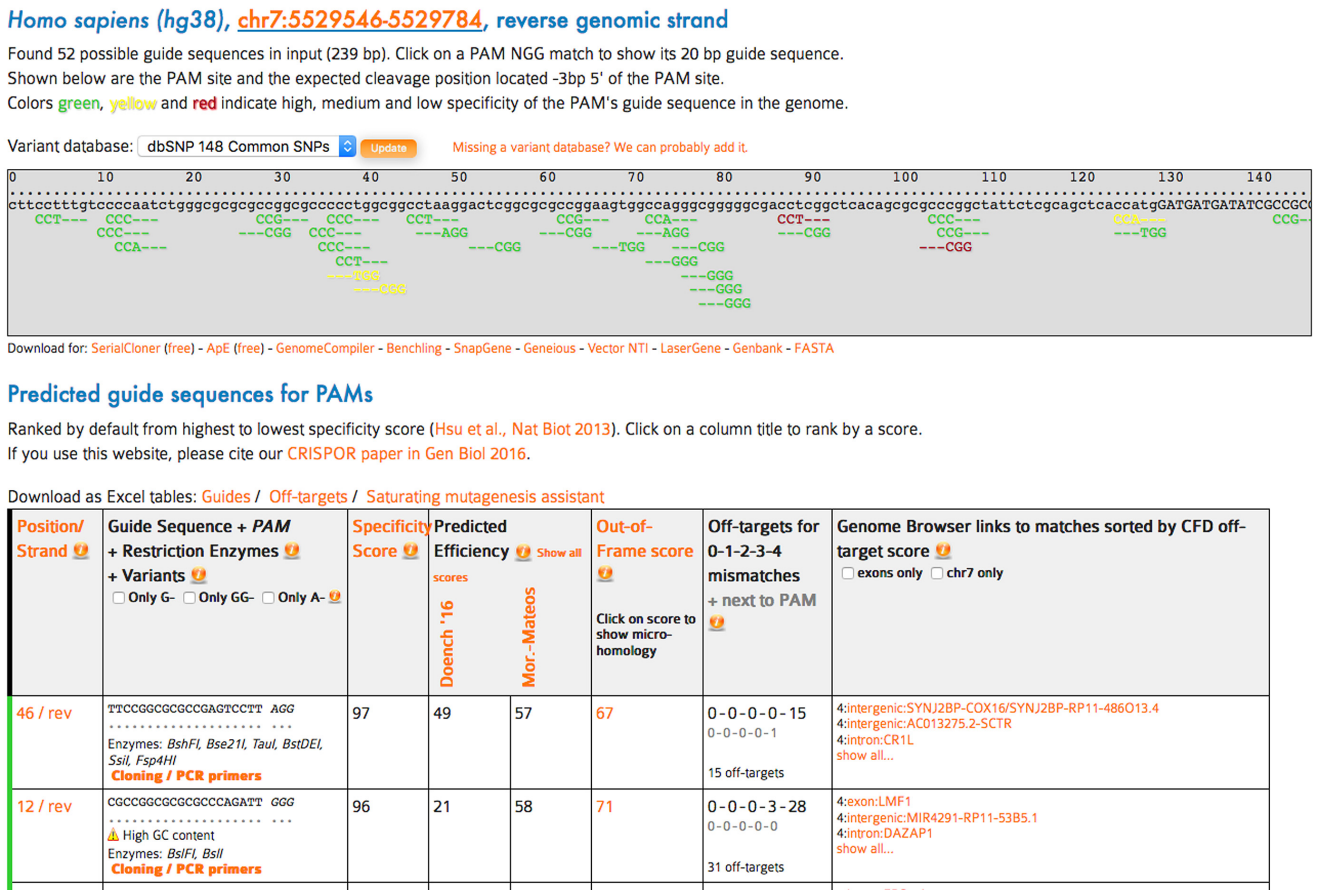
Main output of CRISPOR. At the top, a graphical representation of the input sequence with variants and CRISPR target sequences. Below, a table with guide targets, one per row, annotated with predictive scores, off-target numers and locations and a link the PCR primer designer.

On the input page, the user enters an optional name, pastes a sequence of interest—typically an exon—into the sequence box and chooses a genome and the type of CRISPR nuclease that will be used. Genomes can be searched with the organism's common or scientific name. To keep track of certain sequence locations, the user can mark parts of an input sequence with uppercase and lowercase letters. After a short computation, the website shows a graphical view of the input sequence (Figure 1), with possible guide targets below. This results page is available indefinitely at the moment, and will be for at least one year in the future. Only the PAMs (protospacer adjacent motif) of the targets are highlighted. The strand of the guide is indicated with ‘—’, which corresponds to the location of most short indels induced by SpCas9. Like the original MIT website (http://crispr.mit.edu), guides are colored green, yellow and red, depending on their specificity score (red = avoid, green = recommended). The PAMs can be clicked and link to their respective rows in the table below, which shows the details of a target.

This table contains one row per guide. Initially, rows are sorted by specificity, but can be sorted by any other numeric column by clicking on its title. The first column indicates the position and strand of the PAM, an identifier for this target that is used later to name primers. The second column shows the target sequence, possible restriction enzymes that can be used to screen for successful cleavage and possibly one of three warnings: for very high and very low GC content and whether TTTT is part of the sequence. Guides can be filtered by their first nucleotides (G-, GG-, A-) which is useful with some expression promoters (U6, T7, U3 respectively) and if researchers do not want to add additional, mismatching nucleotides to the 5′end of the guide sequence.

The next three columns show the predictive scores, specificity first, followed by efficiency and out-of-frame scores. These scores are independent: a guide can be predicted to be very specific, but not very efficient or vice versa. Their relative importance of these depends on the context, some projects require specificity first and foremost (e.g. mouse models, clinical applications) others require high efficiency (some cell culture assays) or a high likelihood of out-of-frame mutations (single-guide knock-outs). Users should be aware that at the moment, especially for efficiency, the predictive value of the scores is not very high and will make a visible difference usually when testing more than a few guides. We recommend that users at least try to avoid guides with very low scores. Potential off-targets with up to four mismatches are identified in the selected genome and shown in the last column. Potential off-targets can be filtered to show only ones in exons, which may be more critical in functional studies. In addition, off-targets on chromosomes different from the target chromosome can be hidden, as in animal studies these will not cosegregate with the mutation of interest and will be diluted out.

To select a guide, clicking onto ‘Cloning / PCR primers’ will design overlapping oligonucleotides for guide expression, flanking primers in the genome for validation, and possibly add restriction sites to these, depending on the target AddGene plasmid. The page should be self-explanatory and lists primers organized by the expression method.

## NEW FEATURES

### UCSC Genome Browser tracks

The UCSC Genome Browser provides interactive visualization of genome sequences and extensive annotations including gene exon-intron structures that is essential to design of genome editing experiments. As described in this year's NAR database issue (5), the UCSC Genome Browser now contains a track ‘CRISPR’ that shows pre-computed CRISPOR results for various model organisms and the spCas9 nuclease. The guides are coloured by efficiency score by default, details pages show the predicted off-targets and other scores. A guide can be sent directly to CRISPOR to show more annotations and primers. For computational reasons, the CRISPR guides are only available for exons and ±200 bp around them. Mouse is currently the only exception, the guides cover ±10 kb around exons, so most of the genome. Additional genomes or longer regions can be added by requesting them from the UCSC Genome Browser group. The data files can be downloaded from the UCSC Table Browser or the Genome Browser's download server for processing, e.g. for customized library design. For genomes that do not have a CRISPR track already, or regions that are not yet available on the CRISPR track, any genome sequence can be submitted directly to CRISPOR from the UCSC Genome Browser via the menu ‘View—in external tools—CRISPOR’.

### Batch primer design for off-targets

In general, off-targets predicted by CRISPOR or any other tool are fortunately mostly false positives and only very few will be actually cleaved (4). To find these off-targets, the most sensitive assay is PCR followed by high-throughput amplicon sequencing. But designing dozens of primers can be cumbersome and the analysis of the results is not straightforward. To simplify primer design, we added a feature ‘Off-target primers’ from the ‘Cloning/PCR primers page’ that allow designing one pair of primers flanking every off-target with a single click. For the analysis, we partnered with CRISPResso (6), and output a table in its input format. After PCR and sequencing, the user can upload this table and the FASTQ sequencing file into CRISPResso and obtain histograms with the modification frequency for all off-targets.

### Batch oligonucleotide design for gene knock-out screens and non-coding saturating mutagenesis screens

The most common application of CRISPOR are single guide experiments but increasingly, laboratories are conducting screens in cells using tens of thousands of guides. To make ordering of a library for a subset of several hundreds to thousands of genes easier, a new tool, CRISPOR Batch, accepts a list of genes and outputs a table with oligonucleotides ready to order from oligonucleotide pool suppliers, including subpool barcodes. It uses a list of existing, validated guide sequence libraries collected from the literature, e.g. from (7), which are only available for human and mouse at the moment. CRISPOR Batch is available via a link from the front page. Less experienced users can rapidly obtain guide sequences for a knockout of a specific gene of interest with CRISPOR batch.

A saturating mutagenesis screen can introduce hundreds of mutations in a DNA sequence of typically 1–2 kb using a library of guides (8), but CRISPOR Batch allows only gene identifiers. Therefore, we added the new feature ‘Saturating mutagenesis’ on the guide output page of CRISPOR. The new tool can output all guides fulfilling some minimal criteria (specificity, efficiency) in the current input sequence to a table, adding subpool barcodes and input files for CRISPresso. The assistant is available via a link from the sequence output page.

Both CRISPOR Batch and the saturating mutagenesis assistant are described in detail in an accompanying protocol by Matt Canver *et al*. (9). It includes details about the CRISPOR guide design, wet-lab protocols for cloning and expression methods for screening and subsequent analysis with CRISPResso.

### Efficiency scores for non-spCas9 nucleases

Thanks to the help by the respective authors, we were able to add the SaCas9 efficiency score by Najm *et al*. (10) and the Seq-DeepCpf1 score by Hwang *et al*. (11).

### Genomic variants

For organisms with well-known variant databases, like 1000 Genomes in humans or the Sanger Mouse Strain variants, genome nucleotide variants are shown above the sequence. Variants below a given frequency can be removed with an input box. If a genome does not have a variant database, users can still mark SNPs with *N* characters in the input sequence which will remove guides that overlap *N*s from the table. This information helps to exclude guide RNAs that may be inactive, when the variant affects the PAM, or less efficient, when variants lead to mismatches with the target sequence (9).

### Increased flexibility of basic CRISPOR features

The current version of CRISPOR includes several modifications that have been implemented in response to feedback from users and advances in the predictions of guide activity that are fully listed on the website. Notably, Cpf1 has been added to the nuclease list. Cpf1 differs from Cas9 by producing cohesive rather than blunt DNA ends after DNA cleavage (12) and has recently been shown to favor programmable sequence editing in zebrafish with single stranded DNA donors (13). Cutting frequency determination (CFD) score is now displayed along potential off-targets because it was shown to more reliably predict off-targets (7). Only Doench 2016 and Moreno-Mateos scores are now shown by default since we previously showed that these give the most reliable on-target activity prediction. The Doench 2016 score is recommended when guides are expressed inside cells from exogenous promoters such as U6 whereas the Moreno-Mateos score should be taken into account when using guides transcribed in vitro from the T7 RNA polymerase promoter. CRISPOR results can be dowloaded in different spreadsheet file formats (.tsv or .xls). To facilitate further analysis, results can now also be downloaded in eight different Genbank-like formats for various third party sequence editors. The respective files are adapted to the sequence editor, e.g. by adding colors or special feature types, in order to make the display in the third party tool as convenient as possible. Finally, the PCR primer designer allows users to choose the *T*_m_ and amplicon length optimal for their sequencing method and assay.

### The CRISPOR manual

CRISPOR has been designed to facilitate intuitive utilization in the design and execution of genome editing experiments. Many mouse over descriptions are available on the different screens and links in orange points to specific information or relevant downstream data. Nevertheless, to provide a full overview of the CRISPOR tool, we have added a manual which can be consulted online or downloaded as a PDF file.

## PLANS FOR THE FUTURE

We are planning to add support for base editors. Base editors consist of fusion of Cas9 nickase to cytidine or adenine deaminase that can convert C:G to T:A or A:T to G:C base pairs, respectively. Base editors can only convert bases that are within a small 5′ window of a suitable guide RNA target sequence and appear to possess different specifity rules than Cas9. In order to further streamline the design of genome experiments, we also plan to include tools to assist with design of DNA donors for programmable sequence modifications. Recent studies have demonstrated the improved efficiency of asymmetric single stranded DNA oligonucleotide donors for introducing inserts smaller than 100 nt as well as the great potential of longer single stranded DNA. We are considering better support for CRISPRi/a, notably integration of the efficiency scores by Horlbeck *et al*. (14). We will also continue to rely on feedback from users, CRISPOR has evolved thanks to the feedback from authors of predictive algorithms that helped us integrate their methods and >100 wet-lab scientists who provided feedback.

## DATA AVAILABILITY

The complete source code of the CRISPOR website and a stand-alone command line version is available from GitHub (https://github.com/maximilianh/crisporWebsite/).
